# The Effect of Achieving Serologic Remission on Subsequent Risk of Relapse, End-Stage Renal Disease, and Mortality in ANCA-Associated Vasculitis: A Target Trial Emulation Study

**DOI:** 10.1136/annrheumdis-2022-222439

**Published:** 2022-06-13

**Authors:** Gregory C. McDermott, Xiaoqing Fu, Claire Cook, Catherine Ahola, Brett Doliner, Jennifer Hanberg, John H. Stone, Hyon K. Choi, Yuqing Zhang, Zachary S. Wallace

**Affiliations:** 1 Division of Rheumatology, Inflammation, and Immunity, Brigham and Women’s Hospital, Boston, MA, USA; 2 Clinical Epidemiology Program, Division of Rheumatology, Allergy, and Immunology, Massachusetts General Hospital, Boston, MA, USA; 3 Department of Medicine, Massachusetts General Hospital, Boston, MA, USA; 4 Rheumatology Unit, Division of Rheumatology, Allergy, and Immunology, Massachusetts General Hospital, Boston, MA, USA; 5 Harvard Medical School, Boston, MA, USA

**Keywords:** ANCA, titer, vasculitis, outcomes

## Abstract

**Objective:**

To evaluate the effect of achieving a negative post-induction ANCA assay on the risk of relapse, end stage renal disease (ESRD), and death in ANCA-associated vasculitis (AAV).

**Methods:**

We emulated a target trial using observational data from the Mass General Brigham AAV cohort comparing patients who achieved vs. did not achieve serologic remission (negative ANCA assay) within 180 days of induction. Outcomes were relapse, ESRD, or death within 5 years, obtained from medical records, the US Renal Data System, and the National Death Index. We placed a “clone” of each patient in both trial arms, censored those deviating from their assigned protocol, and weighted each by the inverse probability of censoring. Outcomes were assessed by pooled logistic regression.

**Results:**

The study included 506 AAV patients. The mean age was 61 years (SD 18) and the majority were female (58%), White (87%), MPO-ANCA+ (72%) and had renal involvement (68%). Rituximab (59%) or cyclophosphamide (33%) was most often used for induction treatment. Within 5 years, 81 (16%) died, 51 (10%) had ESRD, and 64 (13%) had relapse. Patients treated to a negative ANCA assay within 180 days had hazard ratio (HR) 0.55 (95%CI 0.38 to 0.81) for relapse and HR 0.87 (95%CI 0.61 to 1.25) for the composite of ESRD or death within 5 years.

**Conclusions:**

In this emulated target trial from a large AAV cohort, achieving serologic remission within 180 days of induction was associated with lower risk of relapse, but no statistically significant difference in ESRD or mortality outcomes.

## INTRODUCTION

Antineutrophil cytoplasmic antibody (ANCA)-associated vasculitis (AAV) is a small to medium vessel vasculitis characterized by disease relapses, increased risk of end-stage renal disease, and excess mortality.[1,2] Most AAV patients have circulating ANCA that target proteinase 3 (PR3) or myeloperoxidase (MPO) and are considered pathogenic.[3] ANCA testing has been a central component of AAV diagnosis since the 1980s,[4,5] but the measurement of ANCA titers after treatment has been a controversial practice.

Using contemporary induction strategies, the majority of AAV patients achieve clinical remission.[6] However, only a proportion achieve concurrent serologic remission with negative serum ANCA assay.[7–10] Research on the clinical utility of post-treatment ANCA measurements has generated conflicting findings, perhaps due to heterogeneous methods that have investigated variable patient groups. Some studies focused on patients with “persistently positive” titers, while others investigated those with rising titers or “re-emerging” ANCA after negative testing.[7–16]

Interest in using ANCA as a biomarker for disease activity stems from its potentially pathogenic role in AAV disease and early studies suggesting that rising ANCA titer may predict disease flare and relapse.[17,18] However, a subsequent meta-analysis found that repeat ANCA testing to identify patients with rising or persistent ANCA titers had limited utility for guiding patient management.[14] Despite those findings, there was a resurgence of enthusiasm for repeat ANCA testing after the adoption of rituximab for AAV induction treatment since rituximab depletes circulating precursors to ANCA-producing immune cells and significantly decreases ANCA titers.[6,19] However, recent research, including observational studies and the MAINRITSAN2 randomized clinical trial have suggested that rising ANCA titers may be specific but imperfect predictors of AAV relapse.[20]

In light of these conflicting data, the impact of achieving a serologic remission on later risk of relapse, ESRD, and death remains unknown. To investigate the association of post-induction ANCA titers with key AAV outcomes, we emulated a target trial using observational data to examine the effect of achieving a serologic remission after treatment on the subsequent risks of relapse, ESRD, and death within 5 years.

## METHODS

### Study Population

We used the Mass General Brigham (MGB) AAV cohort as the data source. The MGB AAV cohort is a retrospective consecutive inception cohort of AAV patients evaluated and treated at a large multi-hospital, healthcare system in the Boston, Massachusetts area. The cohort contains consecutive AAV patients who were diagnosed and received induction treatment between January 1, 2002 and June 30, 2019 identified using a previously described algorithm and confirmed to have AAV by review of electronic health records (EHR).[21] All patients were PR3-ANCA or MPO-ANCA positive; we excluded patients with eosinophilic granulomatosis with polyangiitis. We extracted data on baseline demographics, laboratory testing, and medications from the EHR. The MGB institutional review board approved this study. Consent was waived due to the retrospective nature of the research. Patients and the public were not involved in the design, conduct, reporting, or dissemination plans of this research.

### ANCA Titers

ANCA testing was performed for clinical purposes by enzyme-linked immunoassay (ELISA) and the assay used varied by calendar time and clinical laboratory. We extracted all available ANCA results from the EHR and classified each test as positive or negative using the associated laboratory reference values. We classified borderline results as positive. We considered a patient to be ANCA-negative if they had a negative ANCA assay (e.g., titer below the assay’s borderline or normal level) result within 180 days of treatment initiation, which we defined as the date of initial immunosuppression prescription for AAV.

### Outcomes: End-Stage Renal Disease, Death, and Relapse

The first outcome of interest was relapse (major and minor) within five years of induction treatment (index date). We reviewed the EHR of all patients to identify relapses. We defined relapse as an increase in Birmingham Vasculitis Score Wegener’s Granulomatosis (BVAS/WG) combined with increased immunosuppressive treatment for signs/symptoms of AAV, consistent with prior studies investigating risk factors for AAV relapse.[22] We did not consider an isolated rise in ANCA titer to represent a disease flare.

The second outcome of interest was the composite of ESRD or death within five years of index date. We defined ESRD as (1) requirement of hemodialysis or peritoneal dialysis for >60 days, (2) dialysis until death if the patient died between day 14-60 of follow up, or (3) renal transplant. We obtained data on ESRD and renal transplant from the United States Renal Data System, which is a national registry of ESRD patients, representing an estimated 94% of patients who receive dialysis or kidney transplantation.[23] For ESRD outcome analyses, we excluded four patients who initiated renal replacement therapy >300 days prior to AAV diagnosis for other reasons. Death data were obtained from the National Death Index, a nationwide mortality index run by the Centers for Disease Control.[24] Additionally, we reviewed the EHR of all patients for vital status, ESRD, or renal transplant outcomes not captured in the national databases. We also considered ESRD and death outcomes individually.

### Covariates

We extracted demographic and disease-specific features including age at diagnosis, sex, PR3- and MPO-ANCA type, induction treatment, estimated glomerular filtration rate (eGFR), and comorbidities to calculate a Charlson comorbidity index (CCI) from the EHR.[25] We reviewed each patient’s records to determine disease manifestations and baseline BVAS/WG.[26] CCI was missing on 59 patients. There were no other missing covariate data.

### Statistical Analysis

We emulated a hypothetical clinical trial comparing the 5-year risks of relapse, ESRD, and death in patients who did vs. did not achieve serologic remission within 180 days of induction treatment. Although the goal of the treating providers may not have been to achieve a specific ANCA level, the emulated target trial assesses the impact of potential treatment strategies and minimizes “immortal time” and other biases associated with retrospective data.[27] Because the exposure of interest (i.e., time to “achieving serologic remission”) was the time duration to reach an exposure level, we adopted a “cloning, censoring, and weighting” approach.[28,29] We created two trial arms, one in which patients achieved a negative ANCA assay within 180 days of induction (“Achieved serologic remission”) and one in which patients’ treatment strategy did not result in serologic remission (“Does not achieve serologic remission.”) We created “clones” of each patient and assigned one duplicate to each trial arm. Censoring of a “clone” occurred when it deviated from the assigned protocol. For example, we censored duplicates assigned to the “serologic remission” group if they did not achieve a negative ANCA assay within 180 days. Similarly, we censored duplicates assigned to the “does not achieve serologic remission” group if their ANCA assay became negative within 180 days. Because censoring may lead to selection bias, we weighted each patient by their inverse probability of censoring. Specifically, the denominator was the probability that a duplicate adhered to the assigned arm determined using a logistic regression model, which consisted of baseline age, sex, ANCA type, induction treatment regimen, BVAS/WG, eGFR, and treatment with plasma exchange. This inverse probability of censoring weighting creates two pseudo-populations where group assignment is independent of prognostic factors for the outcomes of relapse, ESRD or death.

For each analysis, follow up time among those not artificially censored ended at the earliest of: event of interest, end of follow-up at MGB (only for relapse), or 5 years after index date. For the relapse and ESRD outcomes, we accounted for the competing risk of death.[30] We fitted pooled logistic regression models for relapse and the composite outcome of ESRD or death, as well as ESRD and death individually. Because the outcomes were rare, the odds ratios generated from the pooled logistic regressions approximate hazard ratios.[31] We calculated 95% confidence intervals for the estimate of the odds ratios and created cumulative incidence curves for each outcome. We performed several subgroup analyses examining the effect of achieving serologic remission on the risk of each outcome by PR3 or MPO-ANCA+ status, renal involvement, initial induction strategy (rituximab- or cyclophosphamide-based), and use of plasma exchange.

We considered a two-sided p-value <0.05 as the threshold for significance, without adjustment for multiple hypothesis testing. Statistical analysis was performed using SAS, version 9.4 (SAS Institute Inc., Cary, NC, USA).

### Sensitivity Analysis

We performed three sensitivity analyses. First, we repeated our main analyses after using a sequential regression method to calculate baseline CCI on the 24 patients missing this baseline data.[32] Second, to test the robustness of the study finding, we repeated the main analysis for all outcomes after extending the grace period (i.e. the time to achieve a serologic remission) from 180 to 365 days. Third, to protect against bias introduced by comparing results from different ANCA testing platforms, we limited the cohort to patients who had ANCA testing performed at Massachusetts General Hospital.

## RESULTS

**Figure 1:**
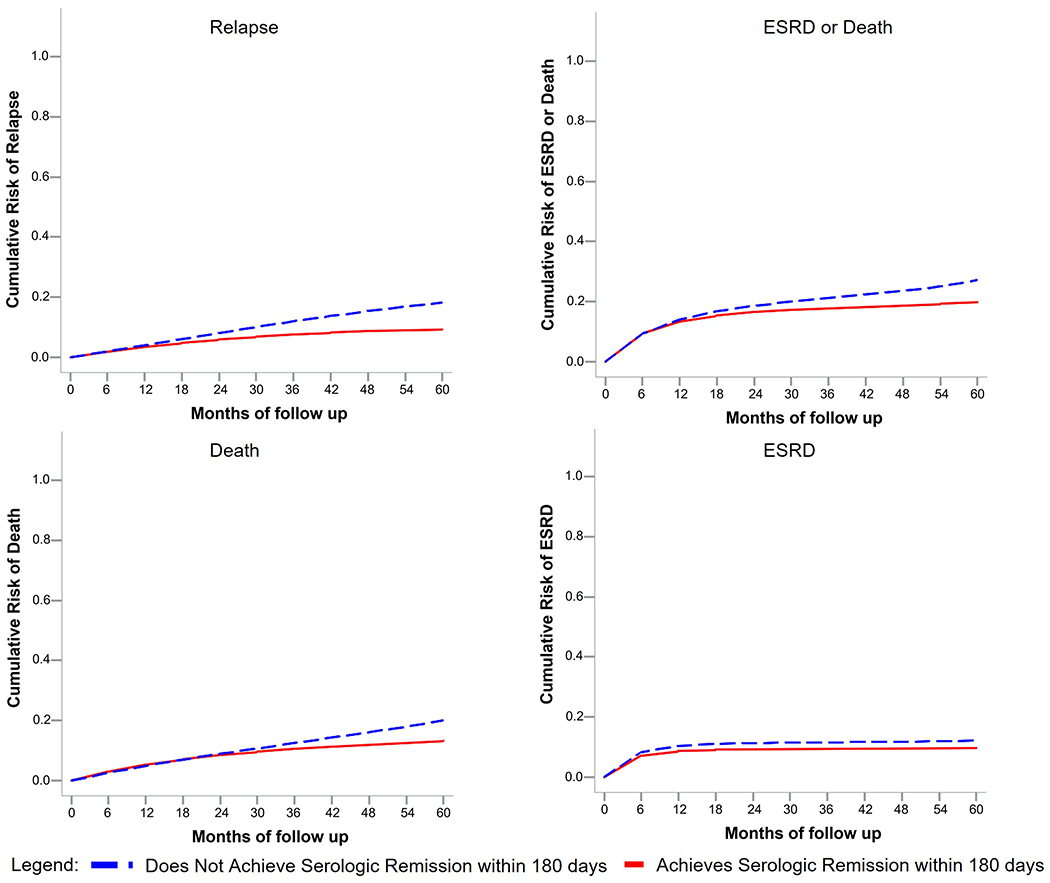
Flow Chart of Eligible Patients and Target Trial Design (180 days)

There were 674 patients in the Mass General Brigham AAV cohort screened for inclusion in the target trial. Figure 1 details patient allocation. After excluding patients lacking ANCA titer measurement within 180 days of induction and/or insufficient baseline information to calculate a CCI, we included 506 patients in this analysis (Table 1). The cohort had a mean age of 61 years (SD 18) and was predominately female (293, 58%), white (442, 87%), and MPO-ANCA positive (366, 72%). Overall, 395 (78%) had major organ involvement at baseline. 342 (68%) had renal and 249 (49%) had pulmonary involvement. The mean baseline BVAS/WG score was 5 (SD 2.2). Induction treatment included primarily rituximab in 298 (59%), cyclophosphamide in 166 (33%), or other treatments (e.g., methotrexate) in 42 (8%). Plasma exchange was used in 119 (24%) patients.

**Figure 2:**
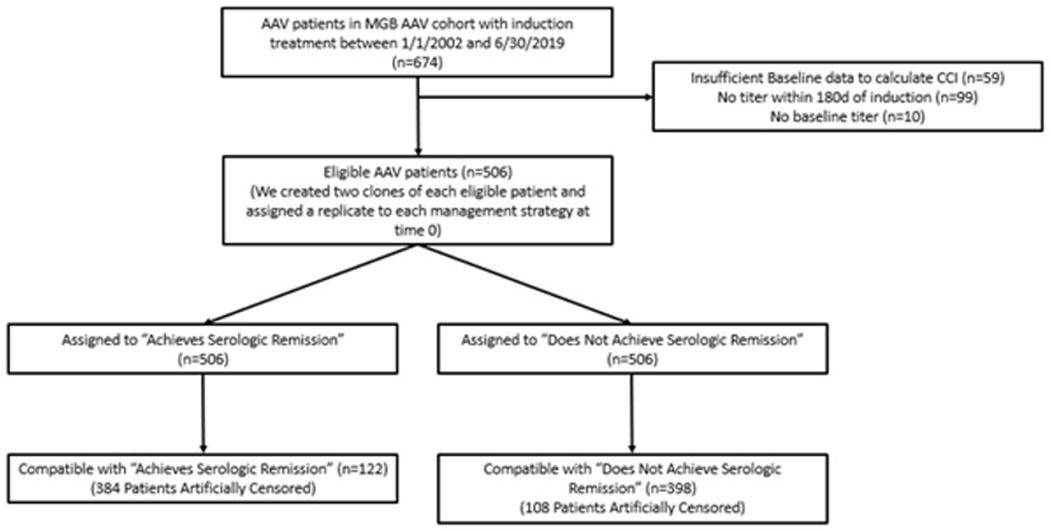
Cumulative Incidence of Relapse, ESRD or Death by Post-Induction ANCA status

The median follow-up time was 49 months (IQR 20.2-60) for relapse and 60 months (IQR 21.9-60) for the assessment of ESRD and death. The mean number of ANCA measurements performed during the first 180 days after induction was 3.5 (SD 1.9). Additional details of the number of ANCA titer measurements overall and in patients with and without major organ involvement at baseline are provided in Supplemental Tables 1–2. During the five years of follow-up, 81 patients (16%) died, 51 (10%) had ESRD, and 64 (13%) had relapse. Among patients who had each outcome, the median time to death, ESRD, and relapse were 675, 33, and 539 days, respectively. Cumulative incidence curves for each outcome are detailed in Figure 2.

In the target trial analysis for relapse, 122 patients achieved a negative ANCA assay within 180 days of induction and were compatible with the “serologic remission” group, while 398 patients were compatible with the “does not achieve serologic remission” group. Censoring of clones in both trial arms is detailed in Figure 1.

The 5-year cumulative incidence of relapse was 9.4 per 100 patients in the group achieving serologic remission and 18.3 in the group that did not achieve serologic remission within 180 days of induction. The corresponding risk difference was −8.9 (95%CI −17.4 to −0.4) per 100 and the HR was 0.55 (95%CI 0.38 to 0.81) (Table 2, Figure 2). Achieving serologic remission was not significantly associated with decreased risk of death or ESRD. The HR for the composite outcome of death or ESRD within 5 years was 0.87 (95%CI 0.61 to 1.25) for the group that achieved a serologic remission.

We observed similar results in subgroup analyses stratifying by ANCA type, baseline renal involvement, and induction treatment (Table 3). Achieving serologic remission within 180 days was associated with a statistically significant reduction in the risk of relapse in the MPO-ANCA+ (HR 0.62 95%CI 0.40 to 0.96) and rituximab-treated groups (HR 0.55 95%CI 0.33 to 0.92). Our sensitivity analyses confirmed the robustness of the findings after imputing data for those with missing baseline CCI, extending the time to achieve a serologic remission from 180 days to 365 days, and limiting ANCA testing to a single laboratory (Table 4).

## DISCUSSION

In this target trial emulation study using observational data from a large cohort of AAV patients, achieving serologic remission (negative ANCA assay) within 180 days of induction was associated with decreased risk of relapse, but was not associated with statistically significant reduction in the risk of ESRD or death within 5 years. We observed similar results when stratifying by ANCA type and induction treatment strategy. These findings suggest that achieving a negative ANCA assay during and after induction may result in fewer subsequent disease relapses.

Our study investigates an ongoing controversy in AAV care that has led to varying ANCA testing practices following diagnosis. Previous studies have yielded conflicting results, in part due to significant heterogeneity of study designs investigating the association of relapses with rise in ANCA titer[11–14], reemergence of ANCA titer[10], ANCA persistence[7–9,14,16], or a combination[20]. Many studies were also conducted prior to the introduction of rituximab, which renewed enthusiasm for ANCA as a clinical biomarker, since rituximab targets the B-cell lineage that ultimately produces ANCA.[19] Recent investigations have suggested that the utility of ANCA titers as a marker of relapse risk may vary by ANCA subtype and specific disease manifestations.[13,16,33]

We expand on these studies using a contemporary cohort of newly diagnosed patients undergoing remission induction, many with rituximab, and applying methods to address immortal time bias and confounding. We focused on the impact of achieving serologic remission (negative ANCA assay) within six months of remission induction. This is an important timepoint in AAV care that typically marks the end of “remission induction” and a transition from induction immunosuppression to maintenance therapy. Achieving a negative ANCA assay at this time may have prognostic significance and inform the choice and intensity of maintenance therapy or subsequent monitoring by identifying patients with favorable AAV treatment response and low risk of subsequent relapse. Our findings remained rather consistent across subgroups stratified by ANCA titer and induction treatment, in contrast to previous studies. Additional studies are needed to evaluate the association of rising ANCA titers with relevant outcomes using similar methodologies to address potential confounding and immortal time bias.

Significant basic and translational research has demonstrated the importance of ANCA for AAV disease pathogenesis. ANCA have been shown to bind to autoantigens and activate neutrophils, leading to microvascular injury.[2] In light of the recognition of the effect that ANCA have on immune cells in animal models and in vitro studies, the inconsistent association between ANCA levels and disease activity remains incompletely understood. Two recent studies suggest that post-translational modification of ANCA immunoglobulins may correlate with differences in disease activity. Espy and colleagues demonstrated that sialylation of PR3-ANCA increased in patients with inactive disease[34] whereas Lardinois and colleagues demonstrated that glycosylation of the Fc segment of IgG was reduced in PR3-ANCA+ patients with active disease.[35] Our findings indicate that, at least in some patients, persistent ANCA beyond remission induction are pathogenic given their effects on relapse risk. However, it is also known that not all patients with a persistent ANCA titer will experience a relapse. More detailed examination of ANCA expression, including post-translational modification, may offer further insights into disease risk in AAV.

Strengths of our study include the use of a large AAV cohort and the assessment of the clinically meaningful outcomes of relapse, ESRD, and death. There has been minimal prior research on the association between ANCA titers and renal and mortality outcomes.[36] We obtained outcome data from comprehensive sources including electronic health record review, the US Renal Data System, and the National Death Index. Another strength was the inclusion of a majority of MPO-ANCA+ patients. Prior literature on the utility of ANCA titers to predict disease flares and outcomes have focused primarily on patients with granulomatosis with polyangiitis who are often PR3-ANCA+.[14] The use of an emulated target trial design with cloning, censoring, and weighting was also a strength of our study. This approach allowed for assessment of the impact of treatment to serologic remission using observational data without the cost of a prospective clinical trial. This technique also leveraged a rich observational dataset while minimizing the effects of immortal time bias, baseline confounding, and selection bias in the weighting step.[37]

Our study has certain potential limitations. First, we relied on observational data from a single healthcare system, which may limit the generalizability of the results. However, the Mass General Brigham system includes community and tertiary care hospitals, primary care, and other specialty clinics throughout many sites in the New England area. Second, we adjusted our analysis for patient baseline factors, but the possibility of residual confounding remains. Third, because we used ANCA test results from multiple reference laboratories and information about the specific assay used was not always available, we were unable to examine if ELISA type impacted the observed associations and directly compare baseline ANCA values between assays.[13] Fourth, we specified a 180 day “grace period” for patients to achieve serologic remission in our target trial, but this window may miss differences between patients who have serologic response early or late within that time period. Fifth, we assessed for relapse outcomes using clinical notes and defined a relapse as intensification of therapy with rise in BVAS/WG score. Although these criteria are agnostic to ANCA titer, the treating providers were not blinded to ANCA results and we cannot account for differences in subsequent treatment and monitoring. Additionally, the relapse rate that we observed was lower than reported in some AAV clinical trials.[6,38,39] This is likely multifactorial, including the MPO-ANCA predominance of our cohort, which has a lower risk of flare[40], as well as the enrollment of patients with relapse into some clinical trials and therefore selection for patients at higher risk of relapse, or other factors. Further prospective studies investigating the effect of achieving serologic remission using structured assessment of disease activity are needed. Finally, we observed an association between achieving serologic remission with decreased risk of relapse and a trend towards decreased ESRD or mortality that did not reach statistical significance. It is possible that our study was underpowered to detect differences in ESRD and death outcomes, which may be long-term consequences of recurrent disease activity. However, our study represents one of the largest published AAV cohorts and was relatively enriched for these outcomes with 23% of subjects experiencing ESRD or death during follow up. Alternatively, significant morbidity and mortality in AAV patients may be less related to disease activity in the modern treatment era. Although we adjusted for induction immunosuppression in our analyses, the induction regimen was not randomly selected and was instead chosen at the discretion of the treating physician based on clinical and other patient factors. Therefore, our findings regarding the prognostic significance of post-induction ANCA titers should not be used to guide clinical management decisions regarding the choice or intensity of induction immunosuppression or subsequent treatment. This represents an important avenue for future prospective research.

In conclusion, we found that achieving serologic remission (negative ANCA assay) during the first 180 days after induction was associated with a decreased risk of relapse within 5 years. We did not observe a statistically significant difference in the risk of ESRD or death within 5 years comparing patients who achieved serologic remission to those who did not achieve serologic remission. We observed similar results after stratifying by ANCA type and induction treatment strategy. These findings suggest that achieving a serologic remission within 180 days of induction is associated with a decreased risk of AAV relapse but may have lower impact on ESRD and mortality outcomes. Further studies are needed to investigate how post-induction ANCA titers and other disease biomarkers may guide AAV management strategies.

## Supplementary Material

Supp1

Supp2

## Figures and Tables

**Table 1:** Baseline Characteristics of Participants (n=506)

Characteristic	Total (n=506, %)
Age (years mean, SD)	61 (18)
Male	213 (42%)
Race	
White	442 (87%)
Black	11 (2%)
Asian	6 (1%)
Other	47 (9%)
ANCA status	
PR3-ANCA+	140 (28%)
MPO-ANCA+	366 (72%)
Organ Involvement	
Any major	395 (78%)
Renal	342 (68%)
eGFR (mL/min/1.72m^2^)	38.8 (14, 72)
Pulmonary	249 (49%)
Head and neck	213 (42%)
Other	66 (13%)
Disease activity at diagnosis (BVAS/WG mean, SD)	5 (2.2)
Charlson Comorbidity Index at diagnosis (CCI mean, SD)	1.7 (2.3)
Induction treatment	
Included RTX	298 (59%)
Included CYC	166 (33%)
Included TPE	119 (24%)
Other (no RTX or CYC)	42 (8%)
Follow up	
ANCA measurements during follow up* (mean, SD)	13 (9)
ANCA measurements within 180 of induction (mean, SD	3.5 (1.9)

* within five years of induction or from induction to relapse or last MGB follow up if <5 years

ANCA = antineutrophil cytoplasmic antibody, BVAS/WG = Birmingham Vasculitis Activity Score for Wegener’s Granulomatosis, CYC = cyclophosphamide, dL = deciliter, eGFR = estimated glomerular filtration rate, mg = milligram, mL = milliliter, mm = millimeter, L = liter, RTX = rituximab, SD = standard deviation

**Table 2:** The effect of achieving serologic remission on risk of relapse, ESRD, and death using an emulated target trial design (n=506)

Outcome	Serologic Remission within 180 days	Persistently Positive Titer at 180 days
*Relapse*		
Risk over 5 years (95% CI), *per 100*	9.4 (3.4 to 15.4)	18.3 (9.9 to 26.7)
Risk difference over 5 years (95% CI), *per 100*	**−8.9 (−17.4 to −0.4)**	Ref
Adjusted HR† (95% CI)	**0.55 (0.38 to 0.81)**	1.0 (Ref)
		
*ESRD or Death (composite)* *		
Risk over 5 years (95% CI), *per 100*	19.8 (11.2 to 28.6)	27.1 (17.0 to 37.3)
Risk difference over 5 years (95% CI), *per 100*	−7.3 (−15.8 to 1.2)	Ref
Adjusted HR† (95% CI)	0.87 (0.61 to 1.25)	1.0 (Ref)
		
*ESRD* *		
Risk over 5 years (95% CI), *per 100*	9.7 (3.6 to 15.8)	12.3 (5.4 to 19.1)
Risk difference over 5 years (95% CI), *per 100*	−2.5 (−11.0 to 5.9)	Ref
Adjusted HR† (95% CI)	0.93 (0.70 to 1.23)	1.0 (Ref)
		
*Death*		
Risk over 5 years (95% CI), *per 100*	13.4 (6.2 to 20.5)	20.0 (11.3 to 28.8)
Risk difference over 5 years (95% CI), *per 100*	−6.7 (−15.1 to 1.8)	Ref
Adjusted HR† (95% CI)	0.81 (0.49 to 1.35)	1.0 (Ref)

* 4 patients with ESRD >300d prior to AAV diagnosis were excluded from analyses of ESRD outcomes

† adjusted for baseline covariates: age, sex, ANCA type, induction treatment regimen, BVAS/WG, eGFR, and treatment with plasma exchange

ANCA = antineutrophil cytoplasmic antibody, ESRD = end-stage renal disease, HR = hazard ratio, CI = confidence interval

**Table 3:** Subgroup Analyses by ANCA Type, Renal Involvement and Induction Treatment Regimen for Relapse, ESRD, and Death

Outcome	Serologic Remission within 180 days HR (95% CI)	Persistently Positive Titer at 180 days
*Adjusted HR*† *for Relapse*		
PR3-ANCA+	0.52 (0.20 to 1.33)	1.0 (Ref)
MPO-ANCA+	**0.62 (0.40 to 0.96)**	1.0 (Ref)
Renal Involvement at Baseline	0.64 (0.39 to 1.03)	1.0 (Ref)
RTX or RTX/CYC treated	**0.55 (0.33 to 0.92)**	1.0 (Ref)
CYC Only treated	0.47 (0.21 to 1.03)	1.0 (Ref)
TPE Treated	0.49 (0.10 to 2.49)	1.0 (Ref)
		
*Adjusted HR*† for ESRD or Death (composite)*		
PR3-ANCA+	0.77 (0.34 to 1.74)	1.0 (Ref)
MPO-ANCA+	0.86 (0.58 to 1.28)	1.0 (Ref)
Renal Involvement at Baseline	0.91 (0.63 to 1.32)	1.0 (Ref)
RTX or RTX/CYC treated	0.98 (0.61 to 1.59)	1.0 (Ref)
CYC Only treated	0.95 (0.55 to 1.64)	1.0 (Ref)
TPE Treated	0.95 (0.64 to 1.40)	1.0 (Ref)
		
*Adjusted HR*† for ESRD*		
PR3-ANCA+	0.55 (0.20 to 1.53)	1.0 (Ref)
MPO-ANCA+	1.03 (0.75 to 1.41)	1.0 (Ref)
Renal Involvement at Baseline	1.00 (0.74 to 1.34)	1.0 (Ref)
RTX or RTX/CYC treated	0.80 (0.53 to 1.21)	1.0 (Ref)
CYC Only treated	1.28 (0.87 to 1.90)	1.0 (Ref)
TPE Treated	0.91 (0.58 to 1.44)	1.0 (Ref)
		
*Adjusted HR*† *for Death*		
PR3-ANCA+	1.04 (0.35 to 3.14)	1.0 (Ref)
MPO-ANCA+	0.73 (0.41 to 1.31)	1.0 (Ref)
Renal Involvement at Baseline	0.88 (0.49 to 1.57)	1.0 (Ref)
RTX or RTX/CYC treated	1.04 (0.55 to 1.96)	1.0 (Ref)
CYC Only treated	0.73 (0.27 to 1.96)	1.0 (Ref)
TPE Treated	1.18 (0.69 to 2.02)	1.0 (Ref)

* 4 patients with ESRD >300d prior to AAV diagnosis were excluded from analyses of ESRD outcomes

† adjusted for baseline covariates: age, sex, ANCA type, induction treatment regimen, BVAS/WG, eGFR, and treatment with plasma exchange

ANCA = antineutrophil cytoplasmic antibody, CYC = cyclophosphamide, ESRD = end-stage renal disease, HR = hazard ratio, MPO = myeloperoxidase, PR3 = proteinase-3, RTX = rituximab, TPE = plasma exchange

**Table 4:** Sensitivity analyses examining target trial outcomes of ESRD, relapse, and death within 5 years

Sensitivity Analysis 1: Imputation of Missing Baseline Data (n=530)*		
	Serologic Remission within 180 Days HR (95%CI)	Persistently Positive Titer at 180 Days
All Patients with imputed baseline data (n=530)*		
Relapse	**0.62 (0.43 to 0.89)**	1.0 (Ref)
ESRD or Death*	0.85 (0.61 to 1.19)	1.0 (Ref)
ESRD*	0.85 (0.67 to 1.06)	1.0 (Ref)
Death	0.79 (0.48 to 1.29)	1.0 (Ref)

Sensitivity Analysis 2: Extending Grace Period to 365 Days (n=506)*		
	Serologic Remission within 365 Days HR (95%CI)	Persistently Positive Titer at 180 Days
All Patients with complete data (n=506)*		
Relapse	**0.73 (0.54 to 0.99)**	1.0 (Ref)
ESRD or Death*	0.94 (0.68 to 1.28)	1.0 (Ref)
ESRD*	0.96 (0.72 to 1.28)	1.0 (Ref)
Death	0.73 (0.46 to 1.17)	1.0 (Ref)
		
Sensitivity Analysis 3: ANCA testing performed at Massachusetts General Hospital		
	Serologic Remission within 180 Days HR (95%CI)	Persistently Positive Titer at 180 Days
All Patients with ANCA performed at MGH (n=453)*		
Relapse	**0.61 (0.40 to 0.93)**	1.0 (Ref)
ESRD or Death*	0.95 (0.65 to 1.39)	1.0 (Ref)
ESRD*	1.00 (0.73 to 1.38)	1.0 (Ref)
Death	0.90 (0.53 to 1.51)	1.0 (Ref)

* 4 patients with ESRD >300d prior to AAV diagnosis were excluded from analyses of ESRD outcomes

ANCA = antineutrophil cytoplasmic antibody, CYC = cyclophosphamide, ESRD = end-stage renal disease, MPO = myeloperoxidase, PR3 = proteinase-3, RTX = rituximab, TPE = plasma exchange

## Data Availability

Data available upon reasonable request and with appropriate institutional review board approval.

## KEY MESSAGES

### What is already known about this subject?

- Anti-neutrophil cytoplasmic antibodies (ANCA) in ANCA-associated vasculitis (AAV) are useful for establishing a diagnosis of AAV and are considered pathogenic.
- The utility of post-induction ANCA titers to inform management or expected outcomes in ANCA-associated vasculitis (AAV) remains controversial.

### What does this study add?

- Using a large cohort of patients with AAV, we performed an emulated target trial comparing patients achieving serologic remission (e.g., negative ANCA assay) to patients who did not achieve serologic remission within 180 days.
- Patients who achieved serologic remission within 6 months of induction treatment had lower risk of relapse by 5 years, but no statistically significant improvement in ESRD or mortality outcomes.

### How might this impact on clinical practice or future developments?

- Treatment of AAV patients to serologic remission may reduce the risk of subsequent disease relapse.
- Future prospective studies should determine the utility of serial ANCA measurements to guide ANCA treatment decisions.
