# Supplementary material for: The Effect of Achieving Serologic Remission on Subsequent Risk of Relapse, End-Stage Renal Disease, and Mortality in ANCA-Associated Vasculitis: A Target Trial Emulation Study
Source: Ann Rheum Dis. Author manuscript; Available in PMC 2023 Oct 1. (PMC9474699; doi:10.1136/annrheumdis-2022-222439)
Supplement: Supp1 [file NIHMS1812273-supplement-Supp1.pdf]

SUPPLEMENTARY MATERIAL FOR: "The Effect of Achieving Serologic Remission on Subsequent Risk of Relapse, End-Stage Renal Disease, and Mortality in ANCA-Associated Vasculitis: A Target Trial Emulation Study" by McDermott, et al

Supplemental Table 1: Mean ANCA titer measurements in AAV patients with and without major organ involvement at baseline

	**Major Organ Involvement at Baseline (n=395)**	**No Major Organ Involvement at Baseline (n=111)**
ANCA measurements during follow up* (mean, SD)	13.1 (8.4)	14.7 (10.4)
ANCA measurements within 180 days of induction (mean, SD)	3.6 (2.0)	3.5 (1.7)

* within five years of induction or from induction to relapse or last MGB follow up if <5 years

Supplemental Table 2: Frequency of ANCA titer testing among AAV patients (n=506)

**Number of ANCA titer measurements**	**During follow up* (n, %)**	**Within 180 days of induction (n, %)**
1	9 (1.8%)	79 (15.6%)
2	22 (4.4%)	94 (18.6%)
3	20 (4%)	106 (21%)
4	35 (6.9%)	87 (17.2%)
5	23 (4.6%)	59 (11.7%)
6	28 (5.5%)	38 (7.5%)
7	24 (4.7%)	23 (4.6%)
8	28 (5.5%)	14 (2.8%)
9	21 (4.2%)	4 (0.8%)
10	14 (2.8%)	1 (0.2%)
11	22 (4.4%)	1 (0.2%)
12	18 (3.6%)	
13	11 (2.2%)	
14	22 (4.4%)	
15	18 (3.6%)	
16	19 (3.8%)	
17	16 (3.2%)	
18	18 (3.6%)	
19	12 (2.4%)	
20	16 (3.2%)	
21	10 (2%)	
22	16 (3.2%)	
23	8 (1.6%)	
24	12 (2.4%)	
25	14 (2.8%)	
26	9 (1.8%)	
27	7 (1.4%)	
28	6 (1.2%)	
29	5 (1%)	

30	3 (0.6%)	
31	5 (1%)	
32	4 (0.8%)	
33	3 (0.6%)	
34	3 (0.6%)	
35	1 (0.2%)	
38	1 (0.2%)	
41	1 (0.2%)	
42	1 (0.2%)	
68	1 (0.2%)	

* within five years of induction or from induction to relapse or last MGB follow up if <5 years
